# Investigation of the effect of hyperthyroidism on endoplasmic reticulum stress and transient receptor potential canonical 1 channel in the kidney

**DOI:** 10.3906/sag-2007-109

**Published:** 2021-06-28

**Authors:** Nuriye Ezgi BEKTUR AYKANAT, Erhan ŞAHİN, Sedat KAÇAR, Rıdvan BAĞCI, Şerife KARAKAYA, Dilek BURUKOĞLU DÖNMEZ, Varol ŞAHİNTÜRK

**Affiliations:** 1 Department of Histology and Embryology, Faculty of Medicine, Atılım University, Ankara Turkey; 2 Department of Histology and Embryology, Faculty of Medicine, Osmangazi University, Ankara Turkey; 3 Department of IVF Unit Andrology Laboratory, Adana City Education and Research Hospital, Adana Turkey

**Keywords:** Hyperthyroidism, endoplasmic reticulum (ER) stress, transient receptor potential canonical 1 (TRPC1), kidney, rat

## Abstract

**Background/aim:**

Hyperthyroidism is associated with results in increased glomerular filtration rate as well as increased renin-angiotensin-aldosterone activation. The disturbance of Ca^2+^ homeostasis in the endoplasmic reticulum (ER) is associated with many diseases, including diabetic nephropathy and hyperthyroidism. Transient receptor potential canonical 1 (TRPC1) channel is the first cloned TRPC family protein. Although it is expressed in many places in the kidney, its function is uncertain. TRPC1 is involved in regulating Ca^2+^ homeostasis, and its upregulation increases ER Ca^2+^ level, activates the unfolded protein response, which leads to cellular damage in the kidney. This study investigated the role of TRPC1 in the kidneys of hyperthyroid rats in terms of ER stress markers that are glucose-regulated protein 78 (GRP78), activating transcription factor 6 (ATF6), (protein kinase R (PKR)-like endoplasmic reticulum kinase) (PERK), Inositol-requiring enzyme 1 (IRE1).

**Materials and methods:**

Twenty male rats were assigned into control and hyperthyroid groups (n = 10). Hyperthyroidism was induced by adding 12 mg/L thyroxine into the drinking water of rats for 4 weeks. The serum-free T3 and T4 (fT3, fT4), TSH, blood urea nitrogen (BUN), and creatinine levels were measured. The histochemical analysis of kidney sections for morphological changes and also immunohistochemical and western blot analysis of kidney sections were performed for GRP78, ATF6, PERK, IRE1, TRPC1 antibodies.

**Results:**

TSH, BUN, and creatinine levels decreased while fT3 and fT4 levels increased in the hyperthyroid rat. The morphologic analysis resulted in the capillary basal membrane thickening in glomeruli and also western blot, and immunohistochemical results showed an increase in TRPC1, GRP78, and ATF6 in the hyperthyroid rat (p < 0.05).

**Conclusion:**

In conclusion, in our study, we showed for the first time that the relationship between ER stress and TRPC1, and their increased expression caused renal damage in hyperthyroid rats.

## 1. Introduction

Triiodothyronine (T3) and thyroxine (T4) are thyroid hormones necessary for normal organ development and metabolic functions [1]. Besides, they regulate growth rate, sodium/potassium pump function, heart rate, blood pressure, respiration, oxygen consumption, digestion, lipid, carbohydrate and protein metabolism, central nervous system function, movements, and metabolic functions of other endocrine glands [2].

Hyperthyroidism has many effects on cholesterol, triglyceride, lipid, oxidation, antioxidants, malondialdehyde (MDA), catalyst enzyme catalase (CAT), liver enzymes, uric acid, urea, creatinine, and histopathological changes in the liver and kidney [3]. Thyroid dysfunction affects kidney physiology and development. In the case of hypothyroidism in which the thyroid gland works less, renal mass (kidney/body mass ratio) decreases, while in the case of hyperthyroidism in which the gland works hard, renal mass increases [4]. However, severe hyperthyroidism results in protein degradation and ultimately renal atrophy. Hyperthyroidism causes an increase in renal blood flow (RBF) and glomerular filtration rate (GFR) [5]. With hyperthyroidism, reabsorption of sodium in the proximal tubule, basolateral Na+/K+ ATPase [6], apical Na+/H+ modifier [7], and Na+/Pi cotransporter [8] activation is increased. Also, the thyroid hormone activates the Na+/Ca^2+^ modifier, possibly via the cAMP-linked pathway, thereby enhancing calcium absorption in the kidneys [9].

Calcium is a second messenger that regulates most of the cellular functions, including gene expression and cellular homeostasis [10], neurotransmitter release and neuronal function [11], metabolism, and modulation of cell life [12]. The transient receptor potential (TRP) channel family is one of the largest families of cation channels. The TRP family is subdivided into TRPC (canonical), TRPM (melastatin), TRPP (polycystin), TRPV (vanilloid), TRPML (mucolip), TRPA (ankyrin), and TRPN (NOMPC-like) subgroups. It is known that TRPC proteins form Ca^2+^ permeable, nonselective cation channels and therefore play an important role in Ca^2+^ signal transduction. The mammalian TRPC family has seven members called TRPC1-TRPC7 [13]. Members of the TRPC family are expressed in different parts of the kidney. TRPC1, which plays a role especially in diabetic nephropathy, is expressed almost everywhere, in renal mesangial cells, glomeruli, proximal tubule cells, and in the thin descending part of the Henle loop, but its exact function is still unknown [14].

Severe Ca^2+^ disorders may trigger cell damage caused by endoplasmic reticulum (ER) stress in response to various pathological conditions [5,15]. Disorders in Ca^2+^ homeostasis in ER are associated with many diseases [16]. ER is an intracellular organelle that is important for regulating Ca^2+^ homeostasis and protein synthesis and folding. Modulation of the expression/function of Ca^2+^ permeable channels also influences intracellular Ca^2+^ concentrations and consequently Ca^2+^ related processes such as cell proliferation, ER stress, apoptosis, and autophagy. ER stress is a condition that disrupts redox balance and luminal Ca^2+^ homeostasis, causing accumulation of unfolded/misfolded proteins. The activation of unfolded protein response (UPR) causes an increase in glucose-regulated protein 78 (GRP78), the ER chaperone, which increases the protein folding capacity of the ER [17]. Activation of ER stress initiates the evolutionarily conserved UPR by the three major signal transducers of ER membrane: PERK, IRE1, and ATF6. Cellular dysfunction and cell death occur under conditions where the ER stress is chronically prolonged and the protein load on the ER substantially exceeds. It is known that one of the pathways causing cell death observed in chronic diseases such as hypoxia, ischemia/reperfusion injury, neurodegeneration, heart disease, and diabetes is due to ER-stress [12].

In light of all this information, we aimed to investigate the relationship between the pathways of TRPC1 signal transduction and ER-stress in kidneys, which are affected generally in hyperthyroidism.

## 2. Materials and methods

### 2.1. Animals

Our study was approved by the Eskişehir Osmangazi University Local Animal Ethics Committee (decision no. 634 in the meeting no. 117 on 30.11.2017) and followed the Guide for the Care and Use of Laboratory Animals published by the National Institutes of Health. Twenty adults (7–8 weeks old) Wistar albino male rats, obtained from the Medical and Surgical Experimental Research Center (TICAM), were maintained at 24 ± 2 ℃ and 55 ± 5% humidity for 12 hours in a light/dark environment during the experiment. Animals were placed in polycarbonate cages for 2 weeks before the experiment and allowed to adjust to ambient conditions, and then randomly assigned to control and hyperthyroid groups. The control group (euthyroid) was fed with a standard rat feed and tap water during the experiment. The hyperthyroid group was established with a standard rat feed for 4 weeks and drinking tap water containing 12 mg/L thyroxine [18]. At the end of the fourth week, rats were euthanized by intramuscular injection of ketamine (45 mg/kg) and xylazine (5 mg/kg). Immediately, intracardiac blood samples were taken from all animals. Whole kidneys were removed by nephrectomy process. Then, the right and left kidneys of rats were cut longitudinally into two pieces. Both right and left kidney pieces were used for both western blot and light microscopy analysis.

### 2.2. Biochemical analyses

Blood samples were centrifuged at 11,000 rpm for 10 minutes. The sera were placed in tubes and stored at -80 ℃ until the day of analysis. Serum-free T3 (fT3), free T4 (fT4), and thyroid-stimulating hormone (TSH) levels were then measured using the ELISA technique according to the manufacturer’s protocol. The Optical Density (OD) levels of expression were read at 450 nm by the BioTek microplate reader 800 (BioTek Instruments, Inc., Winooski, VT, USA), then the results were calculated. The commercial kits for fT3 (YLA0127RA), fT4 (YLA0239RA), and TSH (YLA0047RA) were purchased from Shanghai YL Biotech, China. Also, serum blood urea nitrogen (BUN) and creatinine levels were measured with the absorbance photometry method by Cobas Integra 400 Plus Roche (Roche Diagnostics Ltd., Switzerland).

### 2.3. Histological evaluation

Kidney pieces taken to be examined at the light microscope level were fixed in 10% formaldehyde for 48 h. After routine tissue processing, paraffin blocks were obtained. Then, 5 µm serial sections were taken and stained with the Periodic acid–Schiff technique (PAS). Microscopic evaluation was performed for glomeruli and tubules under an Olympus BX-51 microscope (Olympus America, Inc., New York, USA). Sections were scored in a blinded, semiquantitative manner using an established scoring scale. For each rat in the groups, at least 10 high power (×1000) fields were examined. The percentage of glomeruli that displayed glomerular basement membrane thickening was scored as follows: 0 = none, 1 ≤ 25%, 2 = 25% to 50%, 3 = 50% to 75%, 4 ≥ 75% [19]. The thickness of the glomerular basement membrane (µm) was measured by ZEISS ZEN 3.0 Microscope Software (Carl Zeiss Microscopy GmbH ZEISS Group, München, Germany).

### 2.4. Immunohistochemical evaluation

Paraffin block sections from each kidney tissue were taken onto positively charged slides. After deparaffinization, slides were transferred through degraded ethanol series and then xylol. The sections were incubated with 3% hydrogen peroxide to prevent endogenous peroxidase activity. After antigen retrieval with citrate buffer, the sections were delimited by a pap-pen, washed 3 × 5 min with phosphate buffered saline (PBS), and then blocked with Ultra V-block for 1 h at room temperature. The sections were incubated with rabbit polyclonal ATF6 antibody (ab203119, Abcam Inc., Cambridge, USA), rabbit polyclonal IRE1 antibody (YID5384, Shanghai YL Biotech Co., Ltd, China), mouse monoclonal PERK antibody (sc377400, Santa Cruz Biotechnology Inc., Heidelberg, Germany) and mouse monoclonal TRPC1 antibody (sc133076, Santa Cruz Biotechnology Inc.) overnight at +4 ℃ in humidified conditions. After washing 3 × 5 min with PBS, the sections were incubated with appropriate secondary antibodies (sc2359, sc2781, Santa Cruz Biotechnology, Inc.) for 1 h at room temperature. Then, they were washed again 3 × 5 min with PBS and counterstained with hematoxylin. Slides were applied coverslip using water-based media. Microscopic examination was performed using an Olympus BX51 microscope and computer-assisted imaging system (Olympus America, Inc., New York, USA). 

All immunostained sections were evaluated in a coded manner by the principal author, who was blinded to the group name of experiments. TRPC1, ATF6, IRE1, and PERK expressions were detected mainly in the tubules and/ or glomeruli. The expressions were semiquantitatively determined according to the percentage of positive kidney sections. Staining intensity was classified into four grades: none (0), weak (1), moderate (2), and strong (3). The percentage of positive kidney histological sections was classified as 4 grades: 0 (0%), 1 (1%–10%), 2 (11%–49%) and 3 (50%–100%). The total score was a product of two scores and the final score of one sample was the mean of 10 microscopic fields. TRPC1, ATF6, IE1, and PERK were evaluated according to the reported method [20].

### 2.5. Western blot analyses

To determine the changes in the amount of specific protein in the kidney tissues of hyperthyroidism and control groups, the kidneys were cut into small pieces and homogenization was performed by adding lysis buffer RIPA to 2 mL beaded tubes. After homogenization, the tubes were incubated at 4 ℃ for at least 30 min, and then tissue lysates were centrifuged at 4 ℃, 15,000 g for 20 min. The supernatant containing total protein was transferred into a new tube. Protein determination was performed from the supernatant with Qubit 2.0 device (Invitrogen Inc., Waltham, MA, USA). Fifty µg of protein was loaded into each well, electrophoresed with SDS-PAGE Bio-Rad Mini-Trans Blot (Bio-Rad Laboratories, Inc., California, USA), and then transferred to PVDF membrane with Bio-Rad Trans Turbo device. The membranes were blocked with 5% bovine serum albumin (BSA) for 1 h at room temperature and then, incubated with mouse monoclonal GRP78 antibody (sc166490, Santa Cruz Biotechnology Inc.), rabbit polyclonal ATF6 antibody (ab203119, Abcam Inc., Cambridge, USA), rabbit polyclonal IRE1 antibody (YID5384, Shanghai YL Biotech Co. Ltd., China), mouse monoclonal PERK antibody (sc377400, Santa Cruz Biotechnology Inc.), mouse monoclonal TRPC1 antibody (sc133076, Santa Cruz Biotechnology, Inc.) and mouse monoclonal β-actin antibody (sc47778, Santa Cruz Biotechnology, Inc.) (for loading control) overnight at 4 ℃. Thereafter, the membranes were washed for 3 × 10 min with washing solution (TBST), followed by incubation with appropriate secondary antibodies (sc2005, sc2030, Santa Cruz Biotechnology Inc.). After the membranes were washed for 3 × 10 min with TBST, expression levels of proteins in the immunoreactive bands were monitored by the imaging system (C-Digit, Licor, Cambridge, UK). The image results were analyzed with the Image J 1.49v program.

### 2.6. Statistical analysis

All statistical analyses were performed with IBM SPSS 21.0 statistical package program (IBM Corp., Armonk, NY, USA). The Western blot experiment was repeated three times for each group. The significance level was accepted as p < 0.05. Shapiro–Wilk test was used to determine the normal distribution of the data. The homogeneity of variance test (the Levene test) was used to assess whether groups had equal variances. Independent samples t-test was performed for the data showing normal distribution. Mann–Whitney U test was performed for the data showing non-normal distribution.

## 3. Results

### 3.1. Biochemical results

**Figure 1:**
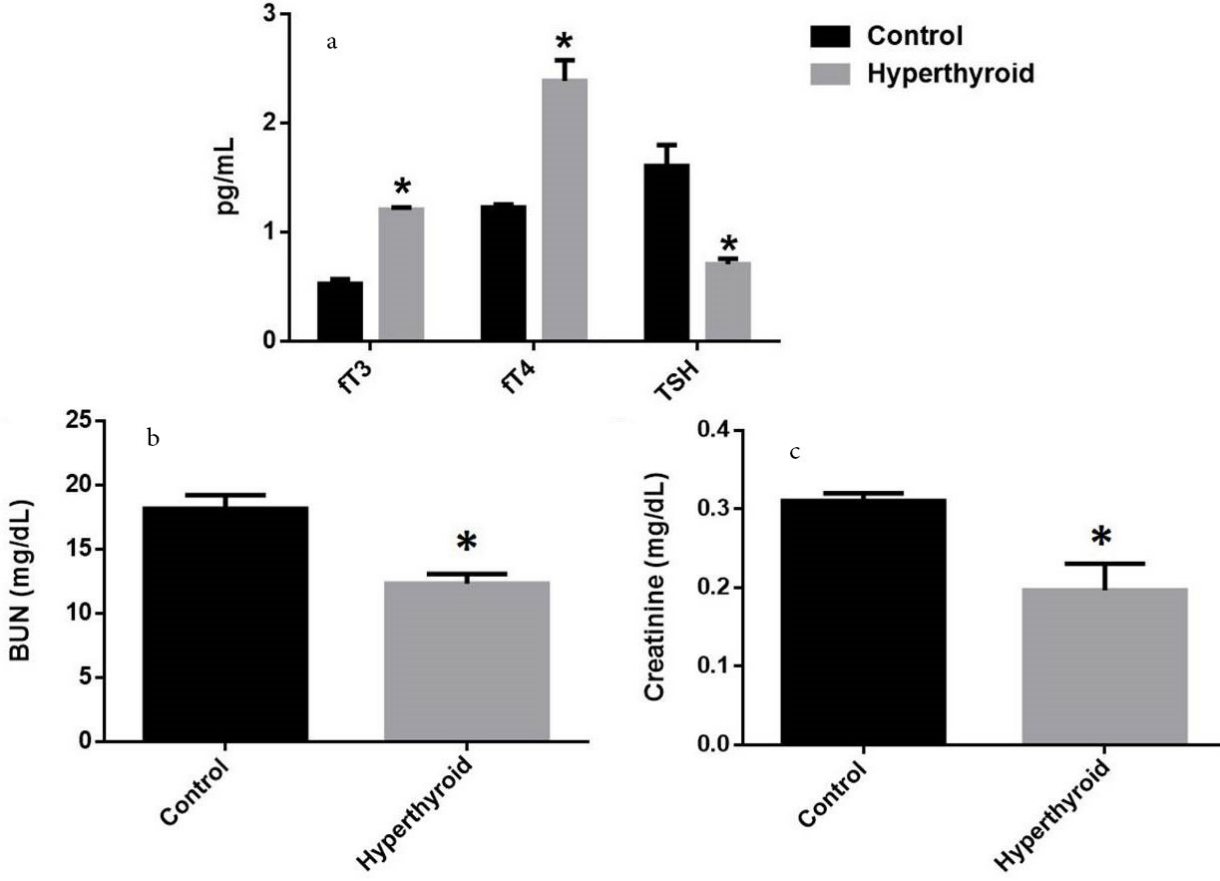
Free triiodothyronine (fT3), free thyroxine (fT4), and thyroid-stimulating hormone (TSH) levels of the rat groups (a). * Different from the control group (p < 0.05). Serum BUN (b) and creatinine (c) levels of the rat groups. *Different from the control group (p < 0.001), an independent samples t-test was performed.

Serum fT3, fT4, and TSH levels, serum BUN, and creatinine levels of the rat groups are shown in Figure 1. Results suggested that fT3 and fT4 levels increased significantly in the hyperthyroid group compared to the control group (both p < 0.05). Besides, a significant decrease was found in TSH level in the hyperthyroid group compared to the control group (p < 0.05) (Figure 1a). Serum BUN (Figure 1b) and creatinine (Figure 1c) levels decreased significantly in the hyperthyroid group compared to the control group (both p < 0.001).

### 3.2. Histochemical results

**Figure 2:**
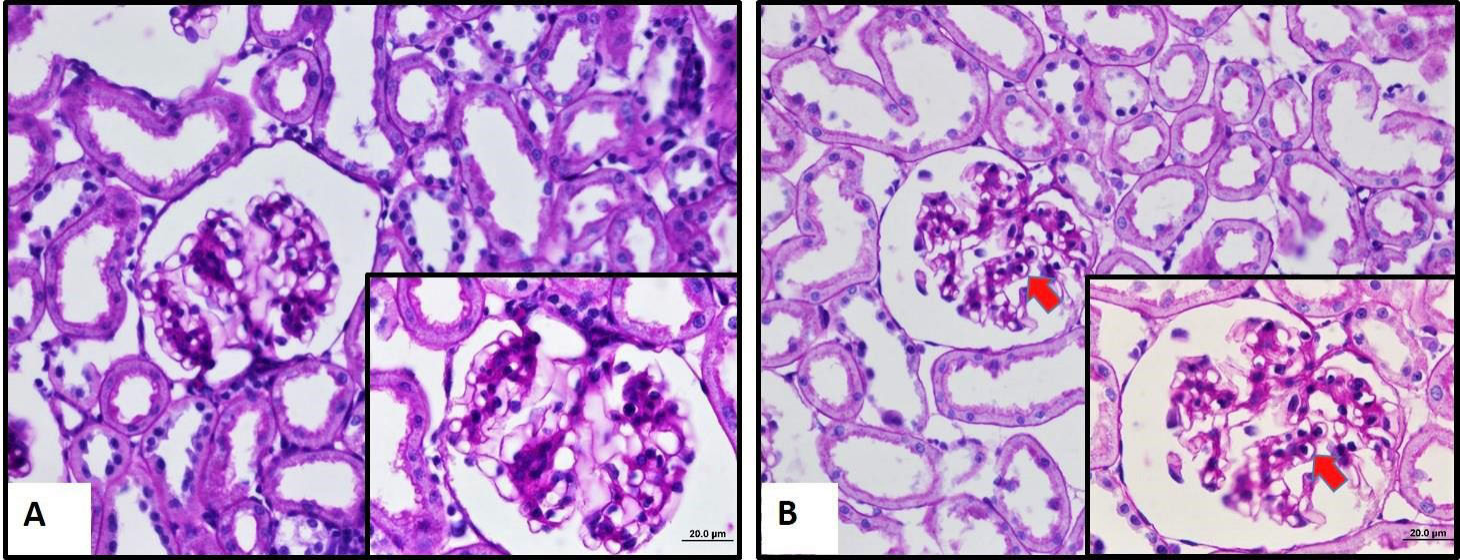
Micrographs of kidney tissues of control (A) and hyperthyroid (B) groups. In the control group, typical kidney histology is seen (A). In the hyperthyroid group, hyperthyroidism caused capillary basal membrane thickening in the glomerulus. Capillary basal membrane thickening in the glomerulus (arrow) was observed (B). There are no obvious differences in the medulla of kidneys in both rat groups. (Periodic acid-Schiff (PAS) Staining). Bars are 20 μm and 50 μm.

**Figure 3:**
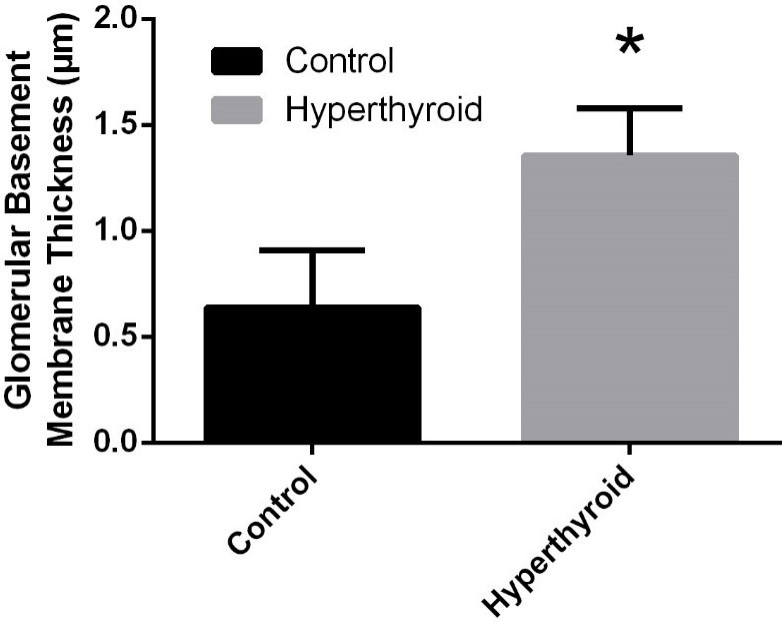
Comparison of glomerular basement membrane thickness between control and hyperthyroid groups. *Different from the control group (p < 0.05), an independent samples t-test was performed.

**Figure 4:**
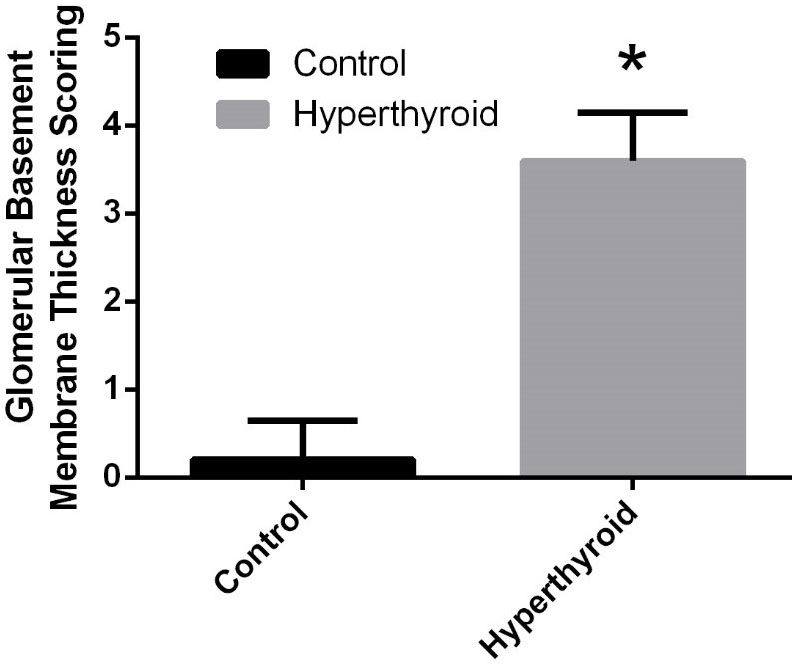
Comparison of glomerular basement membrane thickness scoring between control and hyperthyroid groups. *Different from the control group (p < 0.05), the Mann–Whitney U test was performed.

Histological alterations of kidneys of the rat groups are shown in Figure 2. Kidney histology (the glomeruli, tubules, and vascular structures) was typical in the control group (Figure 2A). However, hyperthyroidism caused capillary basal membrane thickening in the glomerulus (Figure 2B). Glomerular basement membrane thickness increased significantly in the hyperthyroid group compared to the control group (Figure 3–4), (p < 0.05). There was no obvious difference between control and hyperthyroid groups in terms of tubular histology.

### 3.3. Immunohistochemical results

**Figure 5:**
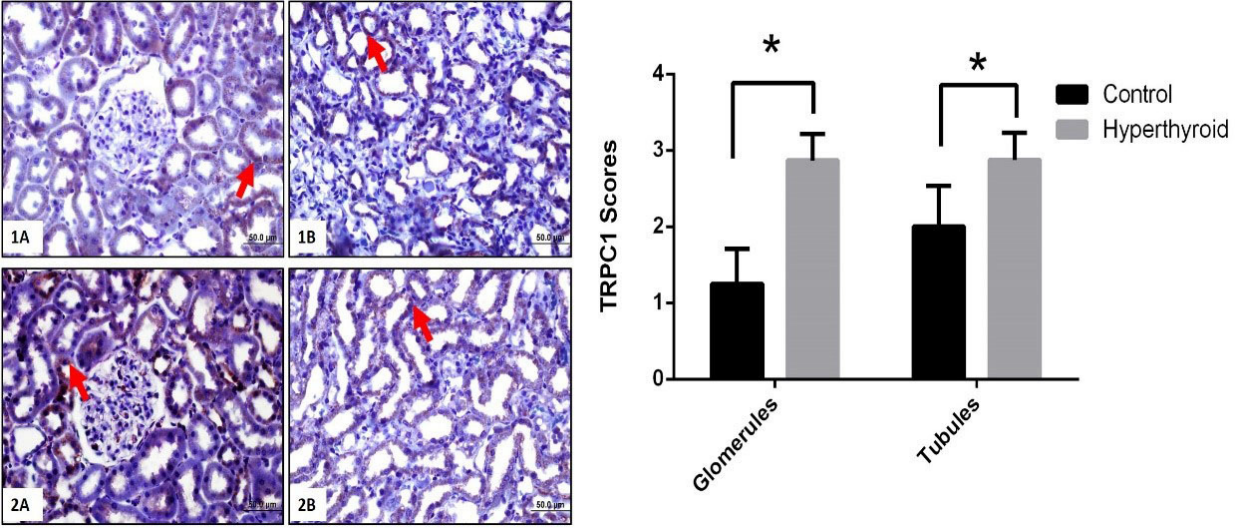
Immunohistochemical staining for TRPC1 in control (1A-B) and hyperthyroid (2A-B) groups in kidney tissue. Expression of TRPC increased in the hyperthyroid group compared to the control group (arrows). Bars are 50 μm. *Different from the control group (p < 0.000), the Mann–Whitney U test was performed.

**Figure 6:**
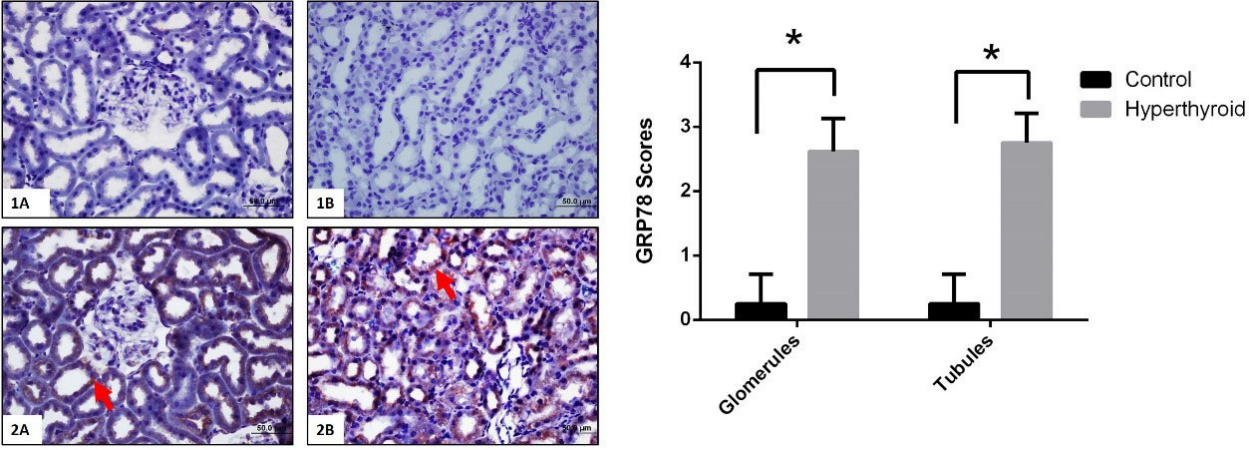
Immunohistochemical staining for GRP78 in control (1A-B) and hyperthyroid (2A-B) groups in kidney tissue. Expression of GRP78 is increased in the hyperthyroid group (arrows). Bars are 50 μm. *Different from the control group (p < 0.000), the Mann–Whitney U test was performed.

**Figure 7:**
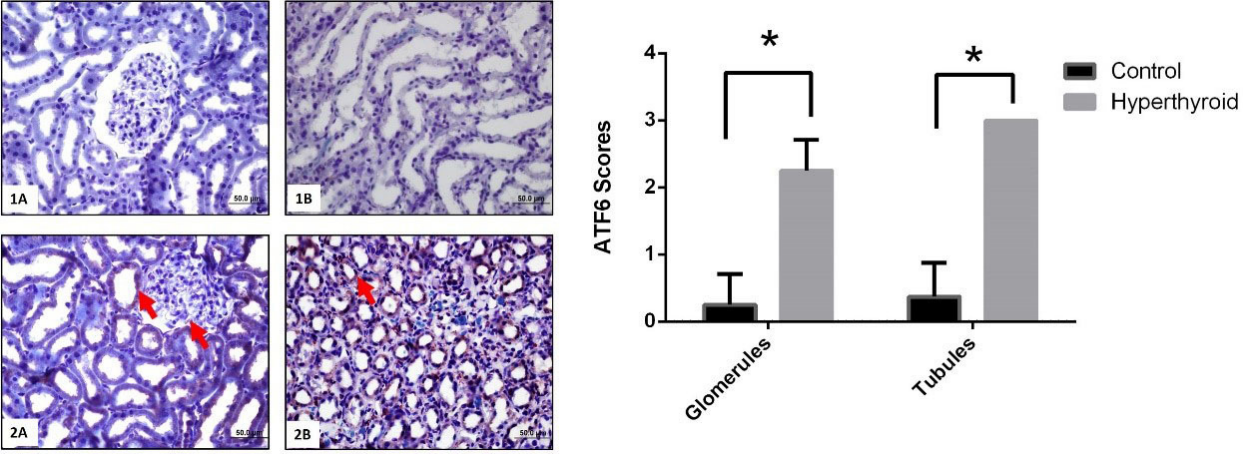
Immunohistochemical staining for ATF6 in control (1A-B) and hyperthyroid (2A-B) groups in kidney tissue. Expression of ATF6 is increased in the hyperthyroid group (arrows). Bars are 50 μm. *Different from the control group (p < 0.000), the Mann–Whitney U test was performed.

**Figure 8:**
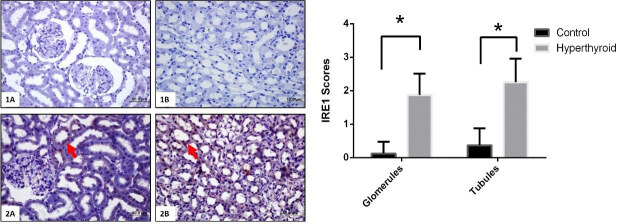
Immunohistochemical staining for IRE1 in control (1A-B) and hyperthyroid (2A-B) groups in kidney tissue. Expression of IRE1 is increased in the hyperthyroid group (arrows). Bars are 50 μm. *Different from the control group (p < 0.000), the Mann–Whitney U test was performed.

**Figure 9:**
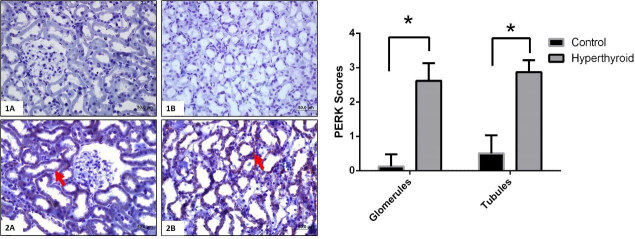
Immunohistochemical staining for PERK in control (1A-B) and hyperthyroid (2A-B) groups in kidney tissue. Expression of PERK is increased in the hyperthyroid group (arrows). Bars are 50 μm. *Different from the control group (p < 0.000), the Mann–Whitney U test was performed.

Immunohistochemical reactions of TRPC1, GRP78, ATF6, IRE1, PERK, and their expressions that are semi-quantitatively determined according to the percentage of positive kidneys of the rat groups (Figure 5–9). Also, their average variation is shown in Table. Compared to the control group TRPC1, GRP78, ATF6, IRE1, and PERK protein expressions significantly increased in the hyperthyroid group in glomerulus and tubules (Figure 5–9) (p < 0,000). Results showed that TRPC1 was expressed in both glomerular and tubular structures in the control group (Figure 5-1A-B) and its expression was increased in the hyperthyroid group (Figure 5-2A-B). GRP78 was not expressed in the control group (Figure 6-1A-B) and its positively reaction was observed in both tubules and glomeruli in the hyperthyroid group (Figure 6-2A-B); whereas, ATF6 was not expressed in the control group (Figure 7-1A-B), its expressions were reacted positively in both tubules and glomeruli in the hyperthyroid group (Figure 7-2A-B). IRE1 was not expressed in the control group (Figure 8-1A-B), and its positively reaction was observed in both tubules and glomeruli in the hyperthyroid group (Figure 8-2A-B). Whereas, PERK was not expressed in the control group (Figure 9-1A-B), the expression of PERK was limited to tubules in the hyperthyroid group (Figure 9-2A-B). 

**Table:** TRPC1, GRP78, ATF6, IRE1, and PERK IHC scorings (p < 0.000), Mann–Whitney U test was performed.

	Glomerulus			Tubulus		
	Control	Hyperthyroid	p value	Control	Hyperthyroid	p value
TRPC1	1.25 ± 0.4629	2.875 ± 0.3535	p < 0.000	2 ± 0.534522	2.875 ± 0.3535	p < 0.000
GRP78	0.25 ± 0.4629	2.625 ± 0.5175	p < 0.000	0.25 ± 0.462	2.75 ± 0.46291	p < 0.000
ATF6	0.25 ± 0.4629	2.25 ± 0.46291	p < 0.000	0.375 ± 0.51	3 ± 0	p < 0.000
IRE1	0.125 ± 0.353	1.875 ± 0.6408	p < 0.000	0.375 ± 0.51	2.25 ± 0.70710	p < 0.000
PERK	0.125 ± 0.353	2.625 ± 0.5175	p < 0.000	0.5 ± 0.534	2.875 ± 0.3535	p < 0.000

### 3.4. Western blot results

**Figure 10:**
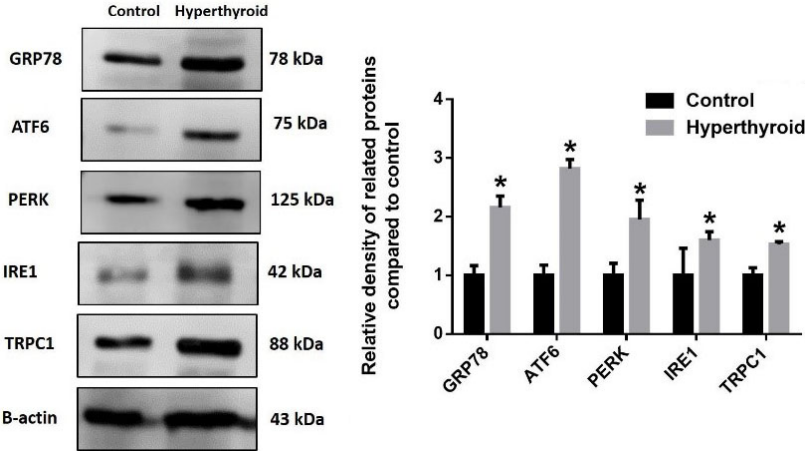
The kidney GRP78, ATF6, TRPC1, IRE1, and PERK protein expression levels and graph of fold change. The increase of all proteins was statistically significant (*p < 0.05), an independent samples t-test was performed.

Western blot results (Figure 10) showed a statistically significant increase in TRPC1, PERK, ATF6, and GRP78 expressions in the hyperthyroid group compared to the control group (p < 0.05). The increase of IRE1 was statistically significant, although not as much as other protein levels (p < 0.05). Hyperthyroidism caused an increase in expression of GRP78 (2.16 fold), ATF6 (2.82 fold), PERK (1.95 fold), IRE1 (1.60 fold), and TRPC1 (1.53 fold) when compared to the control group.

## 4. Discussion

In this study, we suggested for the first time how the renal tissue is affected in hyperthyroidism in terms of the roles of ER stress and TRPC1 channel in this process. In our study, the increased TRPC1 expression caused increment of Ca^2+^ concentration in the cell, in particular, capillary basal membrane thickening and increased GRP78 expression in tubules, especially in glomeruli. We think that ER stress plays an active role in this process of cellular damage, especially through the ATF6 and PERK signal transducer in the UPR (unfolded protein response) pathway.

As it is seen in many studies as in our study [18], fT3 and fT4 values increase in the case of hyperthyroidism, while TSH level decreases. Any dysfunction in the thyroid can affect the production of fT3 and fT4 which can be linked to various pathologies throughout the body [21]. The kidneys play a vital role in the excretion of waste products and toxins such as urea, creatinine and uric acid. Assessment of renal function is important in the management of patients with kidney disease or pathologies affecting renal function. Renal function tests have utility in identifying the presence of renal disease, monitoring the response of kidneys to treatment, and determining the progression of renal disease. Serum creatinine levels decrease in hyperthyroidism, not only due to an increase in GFR but also increment of muscle destruction. Also, the decrease in BUN concentration in hyperthyroidism is thought to be due to decreased expression of aquaporin 1 and 2. Therefore, BUN and creatinine levels decreased in many studies [22] as in our study. Under pathological conditions, cells may lose proteostasis resulting in the accumulation of unfolded and misfolded proteins in the ER, which triggers the UPR or ER stress [23].

Disorders in ER and cytosolic Ca^2+^ homeostasis are associated with many diseases, including the kidney. Disruption of ER Ca^2+^ homeostasis triggers UPR, which prevents further accumulation of newly synthesized proteins in ER. Thus, it shows a prosurvival defense mechanism by reducing the burden on ER [24].

Various factors are known to induce ER stress and trigger kidney damage. ER stress in the kidney may cause tubular epithelial cell differentiation by the mesenchymal transition from the epithelium, renal tubular epithelial cell loss by apoptosis, and ultimately renal pathology including nephron loss, which reduces the filtration capacity of the kidney. In the study of Dickhout et al., ER stress-induced epithelial-mesenchymal transition process in the renal proximal tubule cell line HK-2 for chronic kidney disease was shown [25]. In addition to many studies [9] as seen in our study, hyperthyroidism caused capillary basal membrane thickening in the glomerulus. It was suggested that the increased capillary basal membrane thickening in the glomeruli was formed as a result of the epithelial-mesenchymal transition process due to the increased expression of GRP78 and ER-stress signal converters. The importance of ER stress has been shown in chronic kidney disease in many pathological conditions. In the streptozotocin-induced diabetes model in C57BL/6 mice, ER stress developed and severe nephropathy occurring at the twenty-second month was associated with regulation of CHOP/GADD153, one of the components of the ER stress-mediated apoptosis pathway [26]. In patients with nephrotic syndrome, IgA nephropathy, primary mesangial proliferative glomerulonephritis, and membranous nephropathy, including minimally symptomatic patients, GRP78 and an inducible endoplasmic reticulum (ER) chaperone molecule oxygen-regulated protein 150 (ORP150) immunohistochemical expressions were increased compared to controls. CHOP/GADD153 expression was also increased and nuclear localization was observed in the proximal tubule epithelium of nephrotic patients. In human kidney cells exposed to high levels of serum albumin, ER stress induction was observed and shown to cause apoptosis via the CHOP/GADD153 pathway [25]. The tubulointerstitial ER stress response was found in glomerular diseases with tubular cell apoptosis associated with puromycin amino nucleoside nephrosis, protein overload, and proteinuria associated with experimental or human diabetic nephropathy [27]. Lorz et al. reported that paracetamol, an analgesic, and antipyretic agent, causes renal tubular damage and apoptosis due to ER stress [28]. Paracetamol induces an ER stress response including induction of CHOP and cleavage of caspase-12. Excessive accumulation of secretory proteins induces ER stress, causing podocyte damage [29]. The complement attack also induces ER stress and activates the PERK pathway leading to glomerular epithelial cell damage. All these findings show that ER stress is one of the main causes of kidney damage and the ER stress response is a defense mechanism against kidney damage [30]. In our study, we think that 12 mg/L thyroxine-induced hyperthyroidism causes ER stress in kidney tissue, and the damage caused by hyperthyroidism in kidney tissue is associated with regulation of especially ATF6 and PERK.

The first cloned mammalian TRP channel, the TRPC1 ion channel, is found in the cell within the ER, plasma membrane, intracellular vesicles, and primary ciliary. It expresses almost everywhere in human and rodent tissues. TRPC1 interacts with various protein groups, including ion channel subunits, receptors, and cytosolic proteins to mediate its effect on Ca^2+^ signal. It acts as a nonselective cation channel in pathways that control Ca^2+^ influx in response to cell surface receptor activation. Through this function, proliferation, survival, differentiation, secretion, and cell migration, as well as features such as neuronal growth cones and myoblast fusion of chemotropic cell-specific functions are also available [12].

There are many studies on the change of TRPC1 expression in many pathological conditions causing ER stress. Sukumaran et al. showed that there was a correlation between ER stress and TRPC1 expression that could change cell survival in the salivary gland. They expressed that TRPC1 expression decreased under ER stress conditions and also cell death due to ER stress was dependent on CHOP expression. Increased ER stress and infiltration in salivary gland cells of TRPC1-/- knock-out mice were observed [16].

When kidneys of diabetic rat models were examined, it was reported that TRPC1 mRNA expression was decreased [31]. In patients with diabetic nephropathy or Zucker diabetic rats compared with controls, decreased TRPC1 expression suggested that TRPC1 affected the development of diabetic nephropathy [32]. TRPC1 plays a vital role in the maintenance of ER Ca^2+^ homeostasis, and reduced function leads to prolonged activation of the UPR pathway and disrupts activation of AKT, which then leads to neurodegeneration. A decrease in TRPC1 expression was observed in the neurotoxin-induced mouse Parkinson’s disease model. Increased expression of TRPC1 increases the survival of neurons by regulating the AKT/mTOR pathway, despite ER stress and UPR caused by neurotoxin [33]. Li et al. reported that TRPC1 expression was also increased in Namptin-induced cardiomyocyte hypertrophy depending on time. When the TRPC1 gene was silenced, it resulted in inhibition of nicotinamide phosphoribosyl transferase -induced cardiac hypertrophy. However, by overexpression of TRPC1, nicotinamide phosphoribosyl transferase was thought to induce cardiomyocyte hypertrophy by an ER stress-induced pathway. From this point of view, silencing of the TRPC1 gene may play a protective role in the prevention of cardiac hypertrophy [34]. In our study, ER stress and TRPC1 also increased significantly. This suggests that capillary basal membrane thickening caused by increased ER stress in renal tissue due to hyperthyroidism is due to increased expression of TRPC1.

This study has some limitations. Genetically modified mice might have been used instead of the thyroxine-induced hyperthyroidism rat model. With urine collected using metabolic cages, some kidney function tests might have been measured also in urine. The change in ER stress level could be investigated using the TRPC1 inhibitor.

As a result of our literature review, the effect of hyperthyroidism on the relationship between ER stress and TRPC1 channel in renal tissue was first demonstrated in this study. It was determined that hyperthyroidism caused increment of TRPC1 expression that induces ER stress. We suggest that reducing TRPC1 expression may be a potential therapeutic strategy for hyperthyroid-induced kidney and perhaps other organ damage.
